# Supplementary material for: Chromosome-Level Reference Genome of the Beach False Foxglove, Agalinis fasciculata (Orobanchaceae)
Source: Genome Biol Evol. 2026 May 15;18(5):evag116. doi: 10.1093/gbe/evag116 (PMC13221651; doi:10.1093/gbe/evag116)
Supplement: evag116_Supplementary_Data [file evag116_supplementary_data.zip › SupplementaryFigures_Accepted.docx]

**Chromosome-level reference genome of the beach false foxglove*, Agalinis fasciculata* (Orobanchaceae)**

Pedro H. Pezzi^1^, Maribeth Latvis^1,2*^

^1^ Department of Biological Sciences, University of Arkansas, Fayetteville, AR, USA, 72701;

^2^ University of Arkansas Herbarium, University of Arkansas, Fayetteville, AR, USA, 72701.

**List of Supplementary Figures**

**Figure S1.** Nuclear genome statistics and organelle genome annotation of *Agalinis fasciculata*. (a) Scaffold statistics, BUSCO scores, and composition of the chromosome-level genome in a SnailPlot. (b) Sequencing coverage, sum length, and contamination across the genome in a BlobPlot. (c) Plastid genome structure and annotation, with genes colored by functional group. (d) Mitochondrial genome structure and annotation, with genes colored by functional group. For both (c) and (d), asterisks indicate genes containing introns.

**Figure S2.** GenomeScope profile of *Agalinis fasciculata* based on 21-mers. The k-mer frequency distribution shows two peaks, typical of diploid genomes. GenomeScope estimates a genome size of ~2.3 Gb with low heterozygosity (0.15%).

**Figure S3.** Smudgeplot estimation of the ploidy level of *Agalinis fasciculata* based on 21-mers. The dominant AB smudge (0.76) is consistent with a diploid genome.

**Figure S4.** Telomeric repeats identified in the 14 chromosomes of *Agalinis fasciculata* using tidk. All chromosomes show at least one telomeric peak at one or both chromosome ends. Telomeric repeats are indicated by peaks in the histograms.

**Figure S5.** Omni-C contact map of the chromosome-level assembly of *Agalinis fasciculata*. The contact map shows strong intrachromosomal interactions (red diagonal) across the 14 chromosomes.

**Figure S6.** Tissue-specific gene expression in *Agalinis fasciculata*. Tissue specificity (Tau) of gene expression based on RNA-seq data, shown for flower and flower bud tissues combined (a–b) and for the four tissues analyzed separately (c–d). For (a) and (c), genes with Tau ≥ 0.8 were considered enriched.


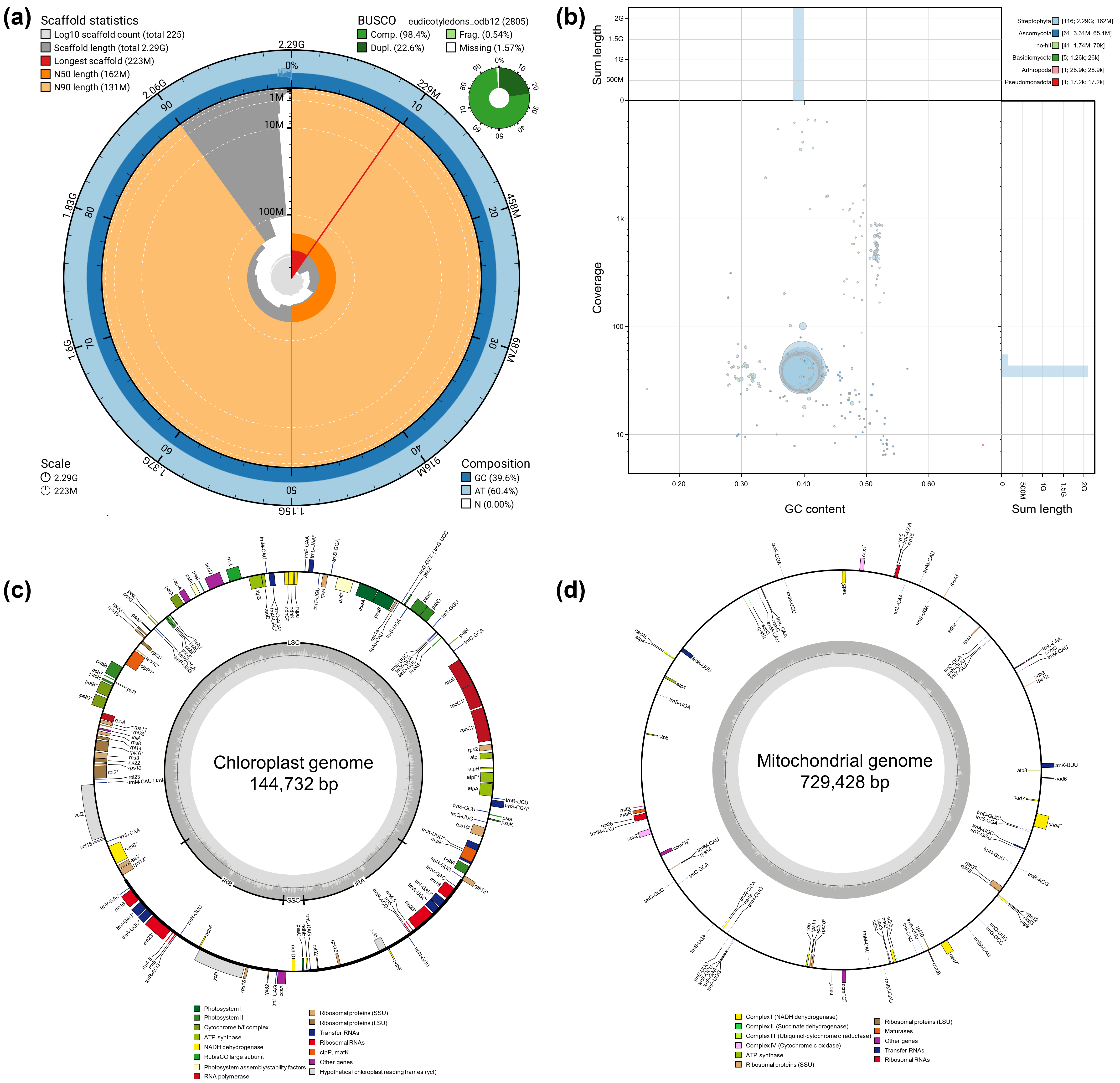


**Figure S1.** Nuclear genome statistics and organelle genome annotation of *Agalinis fasciculata*. (a) Scaffold statistics, BUSCO scores, and composition of the chromosome-level genome in a SnailPlot. (b) Sequencing coverage, sum length, and contamination across the genome in a BlobPlot. (c) Plastid genome structure and annotation, with genes colored by functional group. (d) Mitochondrial genome structure and annotation, with genes colored by functional group. For both (c) and (d), asterisks indicate genes containing introns.

**
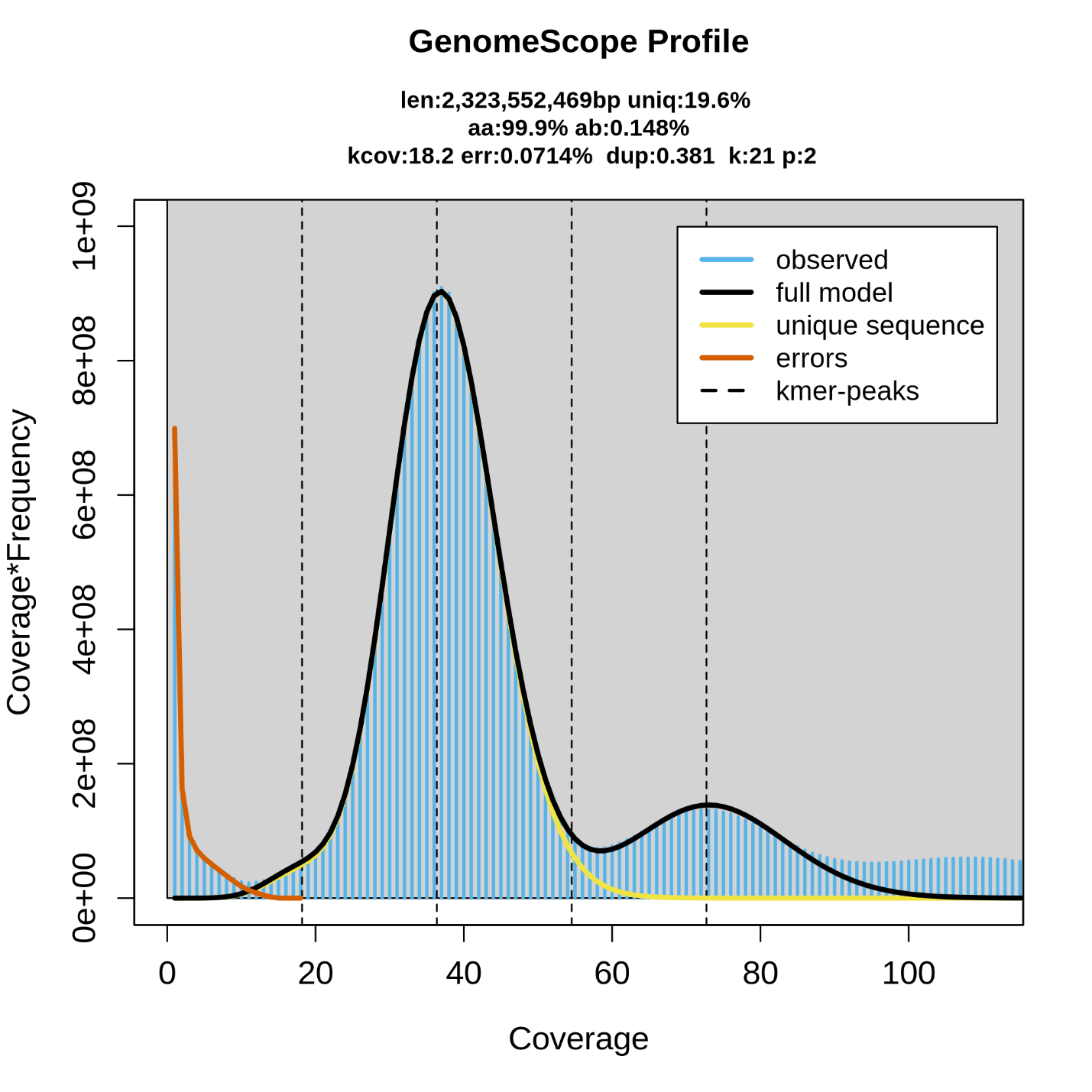
 Figure S2.** GenomeScope profile of *Agalinis fasciculata* based on 21-mers. The k-mer frequency distribution shows two peaks, typical of diploid genomes. GenomeScope estimates a genome size of ~2.3 Gb with low heterozygosity (0.15%).


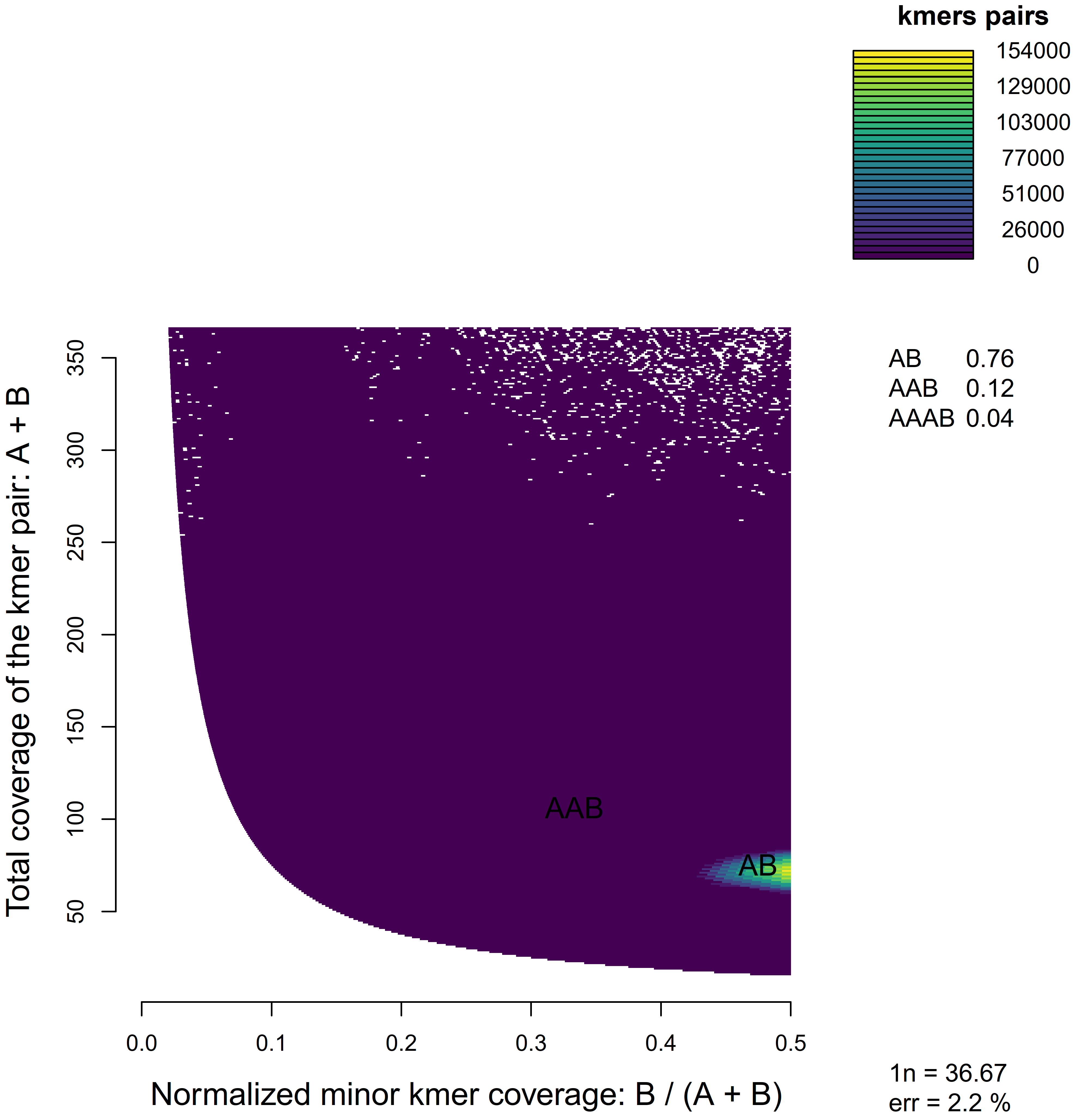


**Figure S3.** Smudgeplot estimation of the ploidy level of *Agalinis fasciculata* based on 21-mers. The dominant AB smudge (0.76) is consistent with a diploid genome.

**
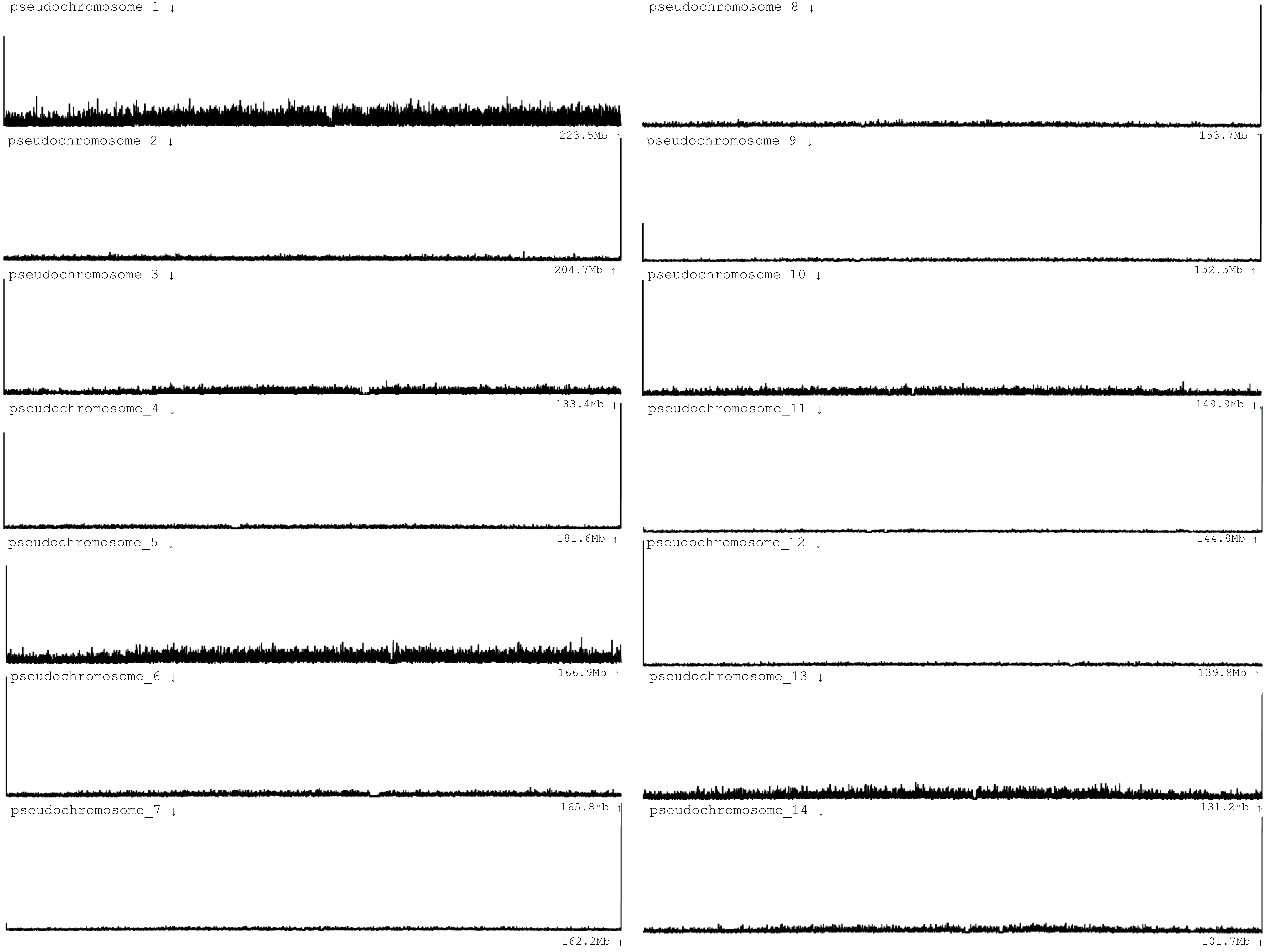
Figure S4.** Telomeric repeats identified in the 14 chromosomes of *Agalinis fasciculata* using tidk. All chromosomes show at least one telomeric peak at one or both chromosome ends. Telomeric repeats are indicated by peaks in the histograms.


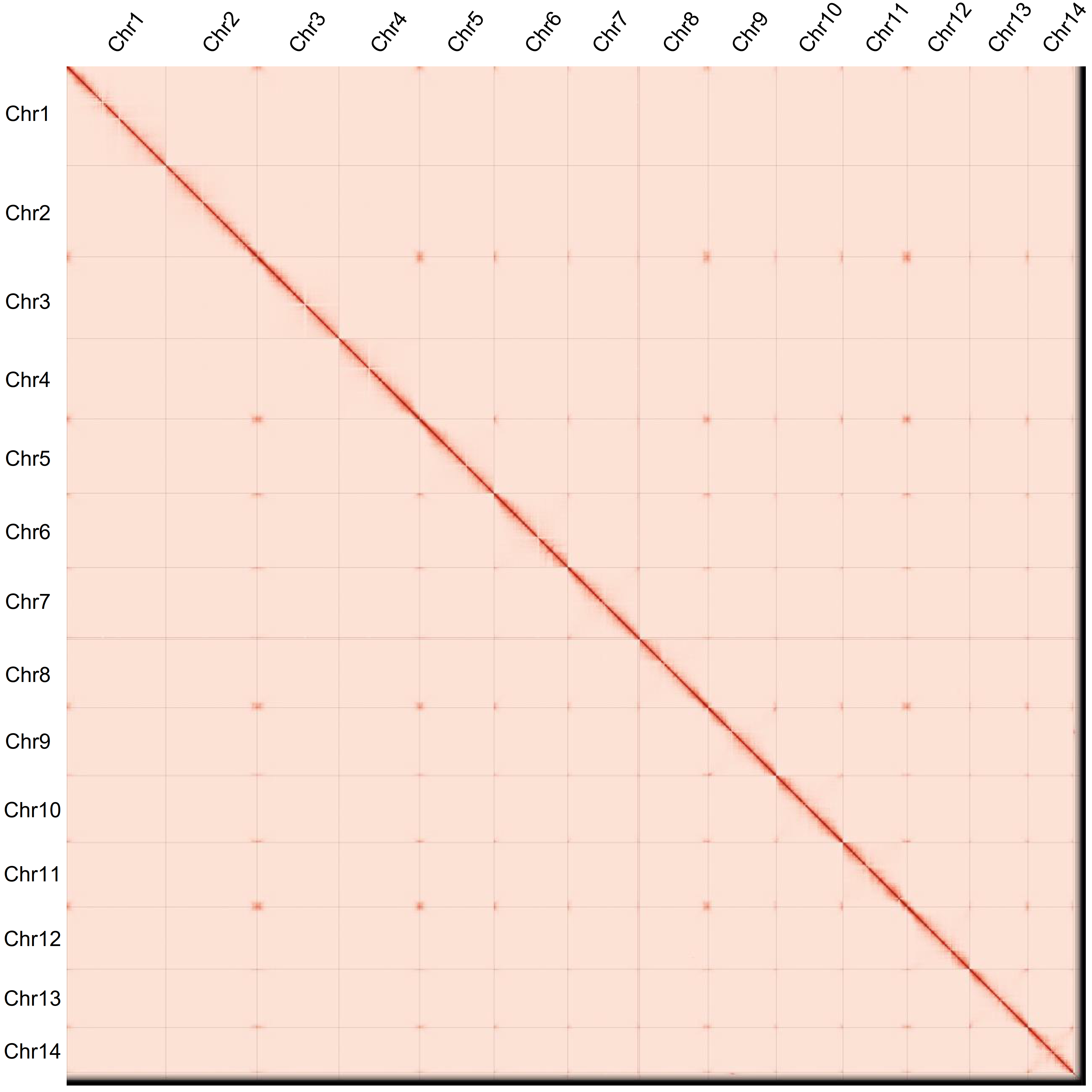
 **Figure S5.** Omni-C contact map of the chromosome-level assembly of *Agalinis fasciculata*. The contact map shows strong intrachromosomal interactions (red diagonal) across the 14 chromosomes.


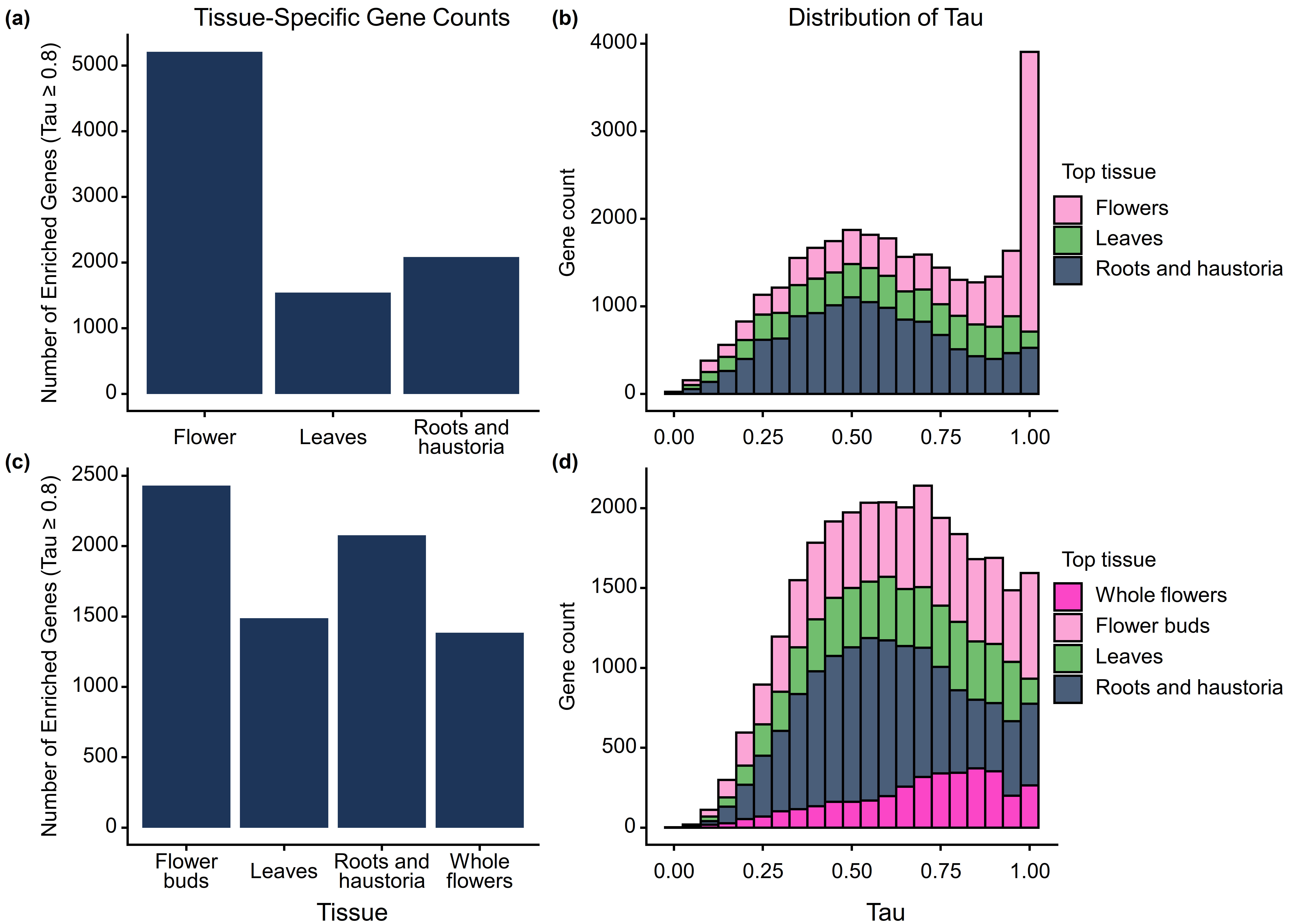


**Figure S6.** Tissue-specific gene expression in *Agalinis fasciculata*. Tissue specificity (Tau) of gene expression based on RNA-seq data, shown for flower and flower bud tissues combined (a–b) and for the four tissues analyzed separately (c–d). For (a) and (c), genes with Tau ≥ 0.8 were considered enriched.
