# Supplementary material for: Chromosome-Level Reference Genome of the Beach False Foxglove, Agalinis fasciculata (Orobanchaceae)
Source: Genome Biol Evol. 2026 May 15;18(5):evag116. doi: 10.1093/gbe/evag116 (PMC13221651; doi:10.1093/gbe/evag116)
Supplement: evag116_Supplementary_Data [file evag116_supplementary_data.zip › SupplementaryTables_R1.pdf]

Chromosome-level reference genome of the beach false foxglove, *Agalinis fasciculata* (Orobanchaceae)

Pedro H. Pezzi[1], Maribeth Latvis[1,2*]

[1] Department of Biological Sciences, University of Arkansas, Fayetteville, AR, USA, 72701;
[2] University of Arkansas Herbarium, University of Arkansas, Fayetteville, AR, USA, 72701.

List of Supplementary Tables

Table S1. Genes present in the plastid (plastome) and mitochondrial (mitogenome) genomes of *Agalinis fasciculata*, grouped by functional category.

Table S2. Number, total length, and genomic proportion of repetitive elements in the *Agalinis fasciculata* genome, as classified by RepeatMasker.

Table S3. Proteomes from Orobanchaceae species used for structural annotation, with corresponding GenBank accession numbers.

Table S1. Genes present in the plastid (plastome) and mitochondrial (mitogenome) genomes of *Agalinis fasciculata*, grouped by functional category.

Genome	Gene group	Gene name
Plastome	Ribosomal RNA	*rrn16*[a], *rrn23*[ab], *rrn4.5*[a], *rrn5*[a]
	Transfer RNA	*trnA-UGC*[ab], *trnC-GCA, trnD-GUC, trnE-UUC*[b], *trnF-GAA, trnG-GCC|trnG-UCC, trnG-UCC|trnS-CGA, trnH-GUG, trnI-GAU|trnE-UUC*[a], *trnK-UUU*[b], *trnL-CAA, trnL-UAA*[b], *trnL-UAG*[a], *trnM-CAU*[a], *trnM-CAU|trnI-CAU, trnN-GUU*[a], *trnP-UGG, trnQ-UUG, trnR-ACG*[a], *trnR-UCU, trnS-GCU, trnS-GGA, trnS-UGA, trnT-GGU, trnT-UGU, trnV-GAC*[a], *trnV-UAC|trnC-ACA*[b], *trnW-CCA, trnY-GUA*
	Small Subunit of ribosome	*rps2, rps3, rps4, rps7, rps8, rps11, rps12*[b], *rps14, rps15*[a], *rps16*[b], *rps18, rps19*
	Large Subunit of ribosome	*rpl2*[b], *rpl14, rpl16*[b], *rpl20, rpl22, rpl23, rpl32*[a], *rpl33, rpl36*
	DNA-dependent RNA polymerase	*rpoA, rpoB, rpoC1*[b], *rpoC2*
	Translational initiation factor	*infA*
	Subunits of photosystem I	*pafI*[b], *pafII, psaA, psaB, psaC, psaI, psaJ*
	Subunits of photosystem II	*psbA, psbB, psbC, psbD, psbE, psbF, psbH, psbI, psbJ, psbK, psbL, psbM, psbT, psbZ*
	NADH oxidoreductase	*ndhB*[b], *ndhC*[d], *ndhD, ndhE, ndhF*[a], *ndhJ, ndhK*[d]
	Subunits of cytochrome	*petA, petB*[b], *petD*[b], *petG, petL, petN*
	Subunits of ATP synthase	*atpA, atpB, atpE, atpF*[b], *atpH, atpI*
	Large subunit of Rubisco	*rbcL*
	Other genes	*accD, ccsA, cemA, clpP*[b], *matK, pbf1*
	Conserved Open Reading Frames	*ycf1*[a], *ycf15, ycf2*
Mitogenome	Ribosomal RNA	*rrn5, rrn18, rrn26*
	Transfer RNA	*trnA-UGC*[b], *trnC-GCA*[a], *trnD-GUC*[a], *trnE-UUC, trnF-GAA*[a], *trnG-GCC, trnH-GUG, trnI-CAU, trnK-UUU*[c], *trnL-CAA*[c], *trnM-CAU*[c], *trnN-GUU*[a], *trnP-UGG, trnQ-UUG, trnR-ACG, trnR-UCU, trnS-GCU, trnS-GGA, trnS-UGA*[c], *trnT-GGU, trnW-CCA, trnY-GUA, trnfM-CAU*[c]
	Small Subunit of ribosome	*rps3*[b], *rps4, rps10*[b], *rps12*[c], *rps13, rps14*[a]
	Large Subunit of ribosome	*rpl5, rpl10, rpl16*
	ATP synthase	*atp1, atp4, atp6, atp8, atp9*
	NADH dehydrogenase – Complex I	*nad1*[b], *nad2*[b], *nad3, nad4*[b], *nad4L, nad5*[b], *nad6, nad7*[ab], *nad9*
	Succinate dehydrogenase – Complex II	*sdh3*[cd], *sdh4*

Ubiquinol-cytochrome c reductase – Complex III	*cob*
Cytochrome c oxidase – Complex IV	*cox1*[b], *cox2*[b], *cox3*
Maturases	*matR*
Other genes	*ccmB*, *ccmC*[a], *ccmFC*[b], *ccmFN*[b], *mttB*

[a]gene with two copies; [b]gene with intron; [c]gene with multiple (>2) copies; [d]fragmented gene (missing start and/or stop codon).

Table S2. Number, total length, and genomic proportion of repetitive elements in the *Agalinis fasciculata* genome, as classified by RepeatMasker.

Class	Order	Superfamily	Number of Elements	Length occupied (bp)	Genome %
**Retroelements**	-	-	977,747	1,676,710,586	73.31
	LINEs	-	38,114	22,465,310	0.98
	LINEs	RTE/Bov-B	9,793	2,646,803	0.12
	LINEs	L1/CIN4	28,321	19,818,507	0.87
	LTR elements	-	939,633	1,654,245,276	72.33
	LTR elements	Ty1/Copia	208,840	546,489,247	23.89
	LTR elements	Gypsy/DIRS1	711,227	1,089,729,195	47.65
**DNA transposons**	-	-	379,953	213,114,117	9.32
	hobo-Activator	-	170,622	75,436,725	3.30
	Tc1-IS630-Pogo	-	49,030	16,351,179	0.71
	MULE-MuDR	-	24,384	17,844,775	0.78
	Tourist/Harbinger	-	47,907	22,297,828	0.97
**Rolling-circles**	-	-	1,771	989,873	0.04
**Unclassified**	-	-	161,288	50,508,830	2.21

Table S3. Proteomes from Orobanchaceae species used for structural annotation, with corresponding GenBank accession numbers.

Species	Accession number
Castilleja foliolosa	GCA_046119335.1
Orobanche gracilis	GCA_050575165.1
Orobanche hederae	GCA_050575145.1
Orobanche minor	GCA_050575125.1
Phtheirospermum japonicum	GCA_014905375.1
Rehmannia glutinosa	GCA_016081115.2
Striga asiatica	GCA_008636005.1
Striga hermonthica	GCA_902706635.1
